# Modeling of leishmaniasis infection dynamics: novel application to the design of effective therapies

**DOI:** 10.1186/1752-0509-6-1

**Published:** 2012-01-05

**Authors:** Bettina M Länger, Cristina Pou-Barreto, Carlos González-Alcón, Basilio Valladares, Bettina Wimmer, Néstor V Torres

**Affiliations:** 1 Grupo de Tecnología Bioquímica. Departamento de Bioquímica y Biología Molecular. Universidad de La Laguna. 38206. San Cristóbal de La Laguna. Tenerife. Spain; 2 Instituto Universitario de Enfermedades Tropicales y Salud Pública de Canarias. Universidad de La Laguna. 38206. San Cristóbal de La Laguna. Tenerife. Spain; 3 Grupo de Tecnología Bioquímica. Departamento de Estadística, I.O. y Ciencias de la Computación. Universidad de La Laguna. 38206. San Cristóbal de La Laguna. Tenerife. Spain

## Abstract

**Background:**

The WHO considers leishmaniasis as one of the six most important tropical diseases worldwide. It is caused by parasites of the genus *Leishmania *that are passed on to humans and animals by the phlebotomine sandfly. Despite all of the research, there is still a lack of understanding on the metabolism of the parasite and the progression of the disease. In this study, a mathematical model of disease progression was developed based on experimental data of clinical symptoms, immunological responses, and parasite load for *Leishmania amazonensis *in *BALB/c *mice.

**Results:**

Four biologically significant variables were chosen to develop a differential equation model based on the GMA power-law formalism. Parameters were determined to minimize error in the model dynamics and time series experimental data. Subsequently, the model robustness was tested and the model predictions were verified by comparing them with experimental observations made in different experimental conditions. The model obtained helps to quantify relationships between the selected variables, leads to a better understanding of disease progression, and aids in the identification of crucial points for introducing therapeutic methods.

**Conclusions:**

Our model can be used to identify the biological factors that must be changed to minimize parasite load in the host body, and contributes to the design of effective therapies.

## Background

The WHO considers leishmaniasis as one of the six most important tropical diseases worldwide [1]. It is caused by parasites of the genus *Leishmania *that are passed to humans and animals by sandflies of the subfamily *Phlebotominae *[2]. Leishmaniasis, which is endemic in 88 countries, has an annual incidence of two million cases and is estimated to cause over 50,000 deaths per year [3]. The disease has three main forms: cutaneous leishmaniasis, mucocutaneous leishmaniasis and visceral leishmaniasis. Visceral leishmaniasis, the most severe form of the disease, is also known as "kala azar", "black fever" or "dumdum fever". It especially affects hosts with weak immune systems, such as children or adults suffering from malnutrition or HIV. The human immune response that limits leishmaniasis is mediated by Th1 cells that activate macrophages to kill the parasite (cellular immunity). When cellular immunity is deficient, an expansion of Th2 cells occurs which allows the parasite to survive within the monocytes and fosters disease development [4]. After an incubation period that varies from ten days to two years [3], typical symptoms are fever, diarrhea, body weight loss, lymphadenopathy, hepatomegaly and splenomegaly. Despite all of the current research, there is still a lack of understanding about the metabolism of the parasite and disease progression.

Mathematical modeling of the processes involved in parasite-host interactions has become a necessary tool in the study of diseases, leishmaniasis being no exception. A significant part of the modeling work in this field is epidemiological [5-7]. In addition, models have also been used to study regulation of gene expression, protein synthesis, and metabolism of the parasite at the genome-wide level [8-10]. The dynamics of parasite-host interactions in the infection process has also been studied using agent-based modeling approaches [11-13]. For example, Dancik et al. [13] used an agent-based stochastic model of the immune response of mice to *L. major *infection to identify parameters that are important in changing the dynamics of the infection process, and to quantify the influence of those parameters. The authors showed that increasing parasite growth rate decreases pathogen load in some circumstances.

There are many studies regarding the biology, epidemiology and immunology of leishmaniasis [5,14,7,15], yet there are fewer studies related to the evolution of the infection in animal models. A significant reference for the latter is the work of Courret et al. [16] where lesion development, cellular response, expression of cytokines, as well as parasite load in the spleen of BALB/c mice infected with *L. amazonensis *is described. In this vein there are also the works of Arrais-Silva et al. [17] on the hypoxia-inducible factor-1 from *L. amazonensis infection*; of Lira et al. [18] on BALB/c mice symptoms, parasite load and immune response in C57BL/6 mice infected with *L. major*; and the work of Requena et al. [19] and of Dea-Ayuela et al. [20] that explores the clinical symptoms, parasite loads and antibody levels in susceptible, oligosymptomatic and resistant hamsters. The study of Requena et al. [19] compared these parameters together with lymphocyte population and proliferation, in two groups infected with different amounts of parasites and a control group.

In the present work we adopted a systems biology approach for understanding disease evolution, host-pathogen interactions, and immune response function. We performed this task by using experimental time series measurements in BALB/c mice infected with *L. amazonensis *to parameterize a mathematical model that accounts for immune response and parasite load. Based on this model we were able to quantify the biological interrelations between variables, perform predictive simulations, carry out sensitivity analysis to evaluate the significance of the system parameters, and solve an optimization problem for minimizing the parasite load. This analysis contributes valuable information to the drug discovery pipeline for developing effective therapeutic methods against leishmaniasis.

## Results

### Mathematical Model

**Figure 1:**
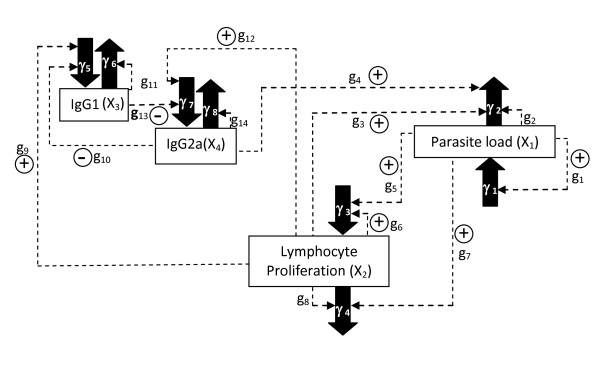
**Leishmaniasis progression model**. Solid arrows represent synthesis (input arrows) or degradation (output arrows) fluxes (each flux number notated by its corresponding γ_i_); dashed arrows are the signals among processes variables which are quantified by the corresponding g_i _value. Positive and negative signs denote activation and inhibition of the corresponding fluxes respectively. See text for the nomenclature.

Experimental measurements obtained in BALB/c mice were used to fit the parameters of the mathematical model shown in Figure 1 that accounts for the progression of Leishmaniasis (see Methods for details). In the present work we used a general power-law formulation, the General Mass Action Power Law formalism (GMA-PL) that allows for non-integer kinetic orders [21,22] with the following structure:

(1) $$v_{i}=\frac{dX_{i}}{dt}=\underset{j}{\sum }\sigma_{ij}\gamma_{j}\underset{k}{\prod }{X_{k}}^{g_{jk}}$$

In the above expression, X_i_, σ_ij_, γ_j _and g_jk _represent the normalized variable set, the stoichiometric matrix, the rate constants, and the kinetic orders, respectively. The variables lymphocytes proliferation (X_2_), IgG1 (X_3_) and IgG2a (X_4_) were normalized with respect to the respective value in the control group of mice. Because the control group is parasite-free, the same approach could not be used to normalize parasite load. In this case the variable was normalized with respect to its own mean value. This standardization reduces the range of variation of the parameters and computation time, and also exploits various properties of the GMA-PL formalism on the behavior of variables and parameters. The specific numerical values for the parameters *σ_ij_*, γ_j _and g_jk _are determined using prior biological knowledge, information about the basal steady-states of the system [21], and/or dynamical experimental data [22,23]. In power-law models, kinetic orders can have non-integer values. One of the main advantages of power-law models is that they allow for the condensation of several steps into simplified representations [21,24,25]. The parameters of the model are kinetic orders and rate constants. Negative values for the kinetic order represent inhibition, that is, an increase in its variable leads to a diminution of the rate involved, while a zero indicates that the variable does not affect the described process. When positive values are considered for a kinetic order, several alternatives are possible: values between zero and one imply a flux that depends on the variable in a saturating-like manner. Values equal to one imply a flux that depends linearly on the variable or, in chemical terms, a first order reaction. By allowing non-integer, positive or negative, kinetic orders, we are able to consider a larger class of kinetic models from which we can select a suitable candidate without changing the (original) model structure.

Figure 1 shows the model scheme of the chosen variables and the influences among them denoted by arrows and parameters. g_1_, ... , g_14 _stand for kinetic orders representing influences on the creation or degradation fluxes (V_i_) of the four variables.

The total parasite load in the host (X_1_) stimulates its immune response. The parasites multiply in macrophages by binary division. The parasite load growth (V_1_) has a nonlinear dependence on the parasite load through the kinetic order g_1_. Increased parasite load leads to a decrease in the proliferation rate of lymphocytes (V_4_); this interaction is represented by the kinetic order g_7 _[26]. Proliferation (or multiplication) of T lymphocytes (X_2_) occurs when naïve T cells are activated by antigens of the pathogen (g_5_) and then differentiated into effector cells (Th1 or Th2) and memory cells. The activation of lymphocytes is an essential event in the production of specific immune responses (both humoral and cellular) against pathogens. Proliferation was measured following the protocol by Monks et al. [27] using the Stimulation Index (see Methods). The lymphocyte proliferation V_3 _is also stimulated by X_2_, through g_6_. Cell mediated effectors enhance X_1 _decay (V_2_); this effect is represented by the positive kinetic order g_3 _[28,29].

The host immune system produces IgG1 (X_3_) and IgG2a (X_4_) antibodies which could be linked to the Th2 and Th1 mechanisms respectively [30,31]. This is represented in our model through a positive influence of X_2 _on the rate synthesis of IgG1 (V_5_) through g_9_, and on the rate synthesis of IgG2a (V_7_) through g_12_. These two immunoglobulins are antagonistic, so each of them has a negative influence on the generation rate of the other, namely X_4 _on V_5 _and X_3 _on V_7_. These effects are represented by the kinetic orders g_10 _and g_13_, respectively [32,33]. The IgG2a influences macrophage activity by stimulating the X_1 _rate decay, V_2_. This interaction is represented in our model by the positive kinetic order g_4_. It is assumed that the transformation rates V_2_, V_4_, V_5 _and V_8 _are proportional to X_1_, X_2_, X_3 _and X_4_. These dependences are represented in the model by the positive kinetic orders g_2_, g_8_, g_11 _and g_14_, respectively.

Given that every variable has an influx and an outflow, the stoichiometric coefficients are 1 and -1 for the synthesis and transformation processes respectively. Model parameters were determined by fitting the model to experimental data from mice using a genetic algorithm as described in the methods section.

Accordingly, the power law model derived from the above scheme is given by:

(2) $$\begin{array}{ccc} \frac{d\;X_{1}}{dt} & =0.1688\cdot X_{1}^{0.5334}-0.0432\cdot X_{1}\cdot X_{2}^{0.0463}\cdot X_{4}^{0.081} & \\ \frac{d\;X_{2}}{dt} & =7.7353\cdot X_{1}^{1.4571}\cdot X_{2}^{0.0227}-6.7737\cdot X_{1}^{1.0049}\cdot X_{2}\\ \frac{d\;X_{3}}{dt} & =6.7417\cdot X_{2}^{1.8413}\cdot X_{4}^{-0.0456}-8.3122\cdot X_{3}\\ \frac{d\;X_{4}}{dt} & =4.3688\cdot X_{2}^{1.9438}\cdot X_{3}^{-0.19}-5.7547\cdot X_{4} \end{array}$$

**Figure 2:**
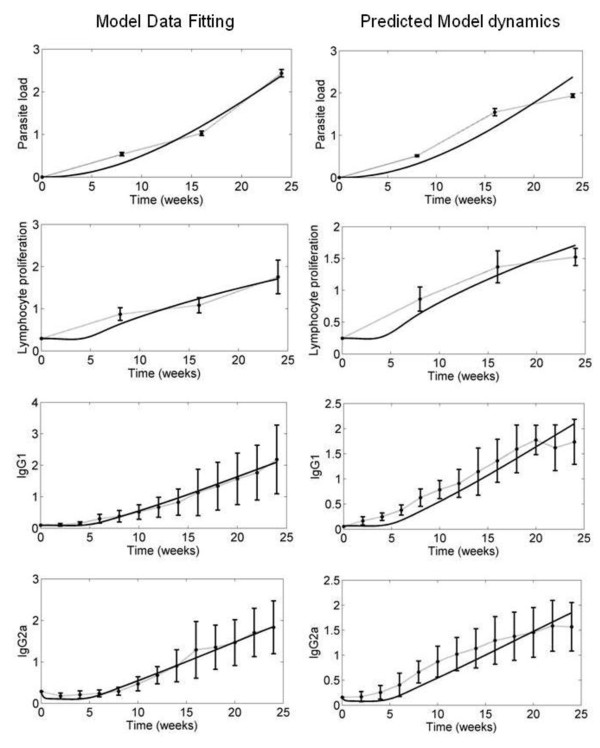
**Data fit and predicted model dynamics of the four model variables**. The panels under Model Data Fitting shows the data fit for the time series data of the four model variables. Panels under Predicted Model dynamics show the comparison of predicted and measured system variable dynamics for an initial parasite load of 10^6^. The continuous lines indicate the estimated dynamics while the dotted lines indicate the experimental data. Error bars indicate the standard deviation among mice.

Figure 2 shows the model data fitting, displaying a good correlation between the experimental and estimated data.

### Model validation

We validated the model by using it to make predictions about the way the system would behave under initial parasite loads that were different from those used to calculate parameter models (106 as compared to 103, see Methods for details). We then performed the corresponding experiments in vivo (see Methods), measuring the four variables described by the model and comparing their observed behavior to model predictions. This initial number of parasites (which mimics a severe leishmaniasis condition) was chosen to check the model capacity to correctly describe the behavior in extreme and differing conditions of initial parasite load. Since the model's main purpose is for the design of therapeutic strategies, a model able to describe a wide range of parasite load dynamics is of foremost interest. Figure 2 shows the results obtained. There it can be seen that the deviation of model prediction from the experimental data is reasonably good during the initial 20 weeks after infection (which means that new treatments are applicable in this time period) with a parasite load of 10^6^. Since the model describes the evolution over the first 20 weeks after infection, the observed discrepancies in the two experimental conditions considered (model fitting and validation) can be deemed as reasonable in light of the associated experimental error. In this regard, we want to stress the fact that in the experimental data used for model verification, other elements of the immune system may be playing a significant role not addressed by the model, but which could be relevant in conditions of massive infection.

### Sensitivity Analysis

**Figure 3:**
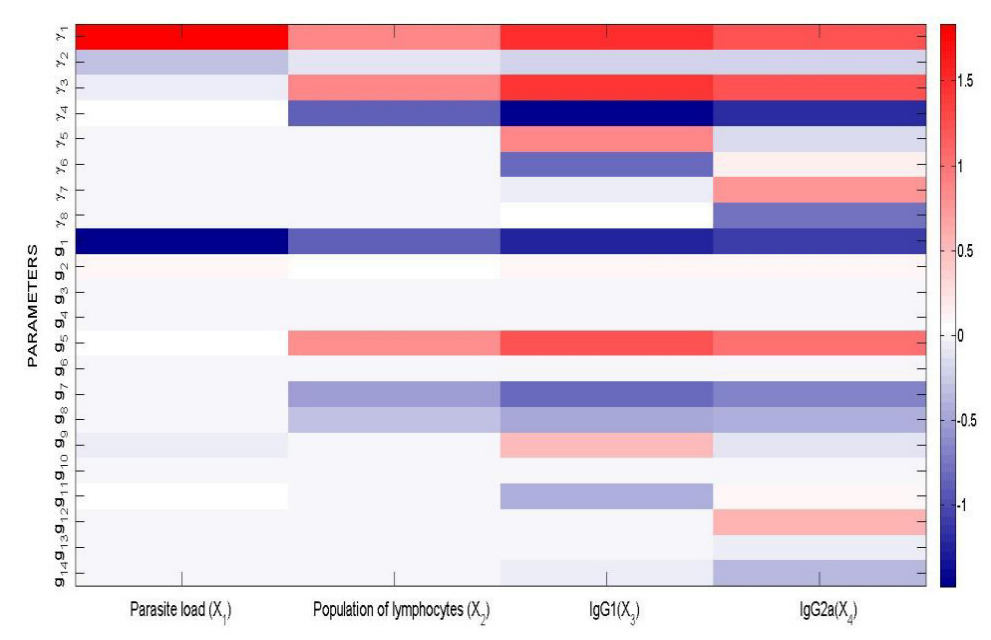
**Absolute value of the system dynamic sensitivities S*_pk_*^Xi ^(X_j _p_k_)**.

Figure 3 shows the dynamic sensitivity analysis for the kinetic orders (g_i_) and rate constants (γ_j_). The System Parameter Dynamic Sensitivity is noted as S*_pk_*^Xi^; *pk *is the parameter under scrutiny and Xi is the considered variable (see equation 6 in Methods). As is shown, all sensitivities have absolute values between 4·10^-5 ^(S(g_10_; X_2_)) and 1.83 (S(γ_1_; X_1_)). The lower value corresponds to the influence of g_10 _on the lymphocyte proliferation, and the higher value measures the influence of the rate constant associated to the parasite multiplication rate on the parasite load. This range of values, together with the observation that the median of the absolute values of sensitivities is 0.067, indicates a robust model.

In general, the higher sensitivities correspond to the variable indicated by the arrow, except for the parameters directly influencing lymphocyte proliferation (γ_3_, γ_4_, g_5_, g_6_, g_7 _and g_8_). For the parasite load, sensitivities with absolute values greater than 1 are S_γ1_^X1 ^and S_g1_^X^. This implies that the generation rate of parasites, and the effect of parasites on their own generation, strongly influence parasite load. Sensitivity S_γ2_^X1 ^is much lower than S_γ1_^X1^. All the other parameters yield sensitivities with absolute values of less than 0.1 for parasite load. The sensitivity of IgG1 and IgG2a to most of the parameters is higher than the sensitivity of lymphocyte proliferation or parasite load to the same parameters. This could be a consequence of the fact that most of the values for parameters directly influencing the parasite load (γ_1_, γ_2_, g_1_, g_2_, g_3_, and g_4_) are small (< 1), as opposed to those directly influencing lymphocyte proliferation.

### Systematic search of parameter profiles for the minimization of parasite load

In order to apply the model for therapeutic purposes, we carried out a systematic search of parameter values that minimize the parasite load. The aim was to discover the set of parameter values (kinetic constants, g_i _and rate constants, γ_i_) that yields a reduced, minimum value of the parasite load, both during the time of infection evolution and at the final, 24-week stage.

The search program was organized in two phases. In the first phase, we changed only one of the parameters at a time (g_i _or γ_i_), with the others maintaining their original values. In this case the value of the candidate parameter is initially set to 10% of the model estimated parameter, the following to 20%, 30% and so on, until the parameter reaches the upper-bound range that was assumed feasible and physiologically relevant. Then, for each changing factor, the model solutions were calculated. In order to evaluate the effectiveness of the change in parameter value, the mean, maximal and final parasite loads were calculated. The mean parasite load reflects the average severity of the disease, the maximal value accounts for the maximal number of parasites along the infection dynamics (which has to be lower than the maximum number of parasites the organism can bear), and the final parasite load represents the final outcome of the disease.

### Single-parameter search for identification of optimum parameter values

#### *g_i _*parameter scanning

**Figure 4:**
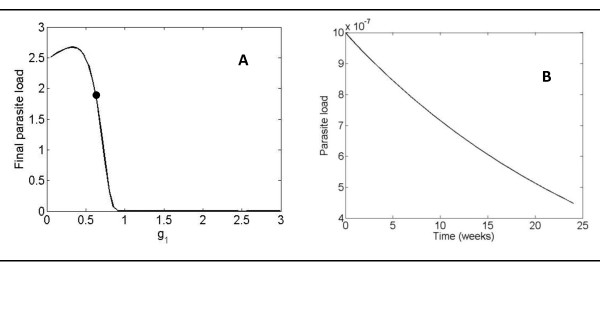
**Evolution of the parasite load over time for different values of g_1_**. A. Final parasite load during a time period of 24 weeks for different values of g_1_. The dot indicates the position of the original system (g_1 _= 0.53). **B**. Parasite load over time for g_1 _= 3.

After a systematic search among all kinetic constants, we found that g_1 _and g_6 _were the most suitable parameters to be changed for reducing parasite load. g_1_, describes the influence of parasites on their own proliferation and is the most significant in this regard, since changes in its value causes the greatest reduction. This is achieved by increasing g_1 _value from 0.53 to 3. Figure 4A shows the progression of parasite load during a time period of 24 weeks for values of g_1 _ranging from 0.01 to 3. Our model suggests that increases in g_1 _from its initial value (0.53) have a therapeutic effect, because they lead to a decrease in parasite load and therefore to healing. It is important to note that for values of g_1 _< 0.5, final parasite load is proportional to g_1_. However, for values of g_1 _> 0.5, final parasite load becomes inversely proportional to the value of this parameter. This latter region includes the actual g_1 _value. This implies that therapeutic strategies should aim to increase g_1_. If decreases are sought, such decreases must be well below 0.5 in order to have a similar effect.

Figure 4B shows that the evolution of the parasite load decreases right from the beginning, almost linearly through the 24-week period.

At first glance this result could appear paradoxical and certainly counterintuitive. But, in fact, the sensitivity of g_1 _with respect to the parasite load (X_1_), S*_g1_*^X1^, is negative (see Figure 2), therefore an increase in g_1 _should lead to a decrease in parasite load. This prediction holds true for the mean parasite load over a time period of 24 weeks. However, it has been observed that both the maximum and the final value of parasite load increase in the beginning until they reach a threshold value and decrease from that point on. Again, such a decrease in parasite load indicates that increasing g_1 _produces a therapeutic effect. The same result has recently been observed by Dancik et al. [13]. Their model showed that increasing parasite growth rate (V_1 _in our model, which is influenced by g_1_) impairs the pathogen load in certain stages of the disease. Since the modeling strategy used by Dancik et al. [13] is different from our approach, there is not a straightforward translation of the parameters. However, there *are *some correlations between their findings and our model predictions. In particular, their analysis [13] indicates that increasing growth rate can, in some circumstances, suppress pathogen loads, which is what our model also predicts. Due to the particular formalism we have used, we are able to point to the increase of g_1 _as the mechanism that increases parasite growth rate. They reported the same evolution pattern in the parasite load that we found: a higher parasite growth rate yields a higher increase in pathogen load in the beginning but also a higher decrease afterwards in such a way that, as a whole, parasites are eliminated earlier. They observed that infection was cleared after eight weeks versus the 17-20 weeks in our model, but this can be attributed to the different *Leishmania *strain and the lower initial parasite load (1000 versus 50). The authors explain this behavior by stating that a pathogen that proliferates rapidly is more likely to be detected by the immune system. Therefore, pathogen load decreases as growth rate increases, with slowly replicating pathogens persisting longer than fast growing ones.

**Figure 5:**
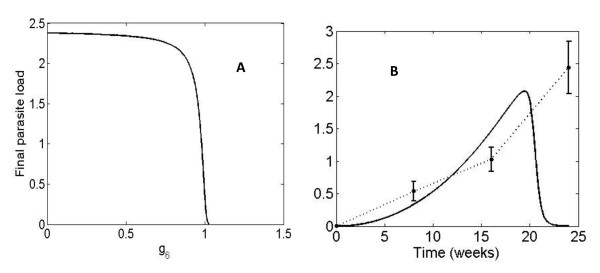
**Evolution of the final parasite load over time for different values of g_6_**. A. Final parasite load during a time period of 24 weeks for different values of g_6_. **B**. Comparison of the predicted evolution of the final parasite load over time for an optimized value of g_6 _(1.02, line) with the experimentally observed parasite load values (dots). In the experimental set-up the estimated value for g_6 _is 0.022.

Another g_i _parameter with similarly minimizing effects on the parasite load is g_6_. g_6 _stands for the influence of lymphocytes on their own production. It has been observed that the parasite load (final, maximal and mean) can be reduced by increasing g_6 _from its original value of 0.02 to 1.02 (Figure 5). This figure shows that parasite load first increases until reaching a maximum after 18 to 20 weeks and then abruptly decreases. The effect of the augmentation of g_6 _could be related to system immune response enhancement. Lymphocytes need some time to identify the pathogens, thus there is a time lag between the start of immune response, the identification of parasites, and their elimination. Accordingly, the parasite load augments until reaching a point where it suddenly decreases.

#### *γ_i _*parameter scanning

**Figure 6:**
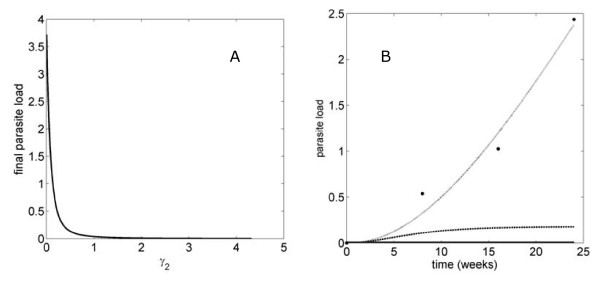
**Evolution of the parasite load over time for different values of γ_2_**. A. Final parasite load during a time period of 24 weeks for different values of γ_2_. **B**. Parasite load over time for (γ_2_)_original _(0.043, dotted line); 0.43 (discontinuous line); 4.3 (continuous line) and experimental data (dots).

We also carried out a systematic search among all rate constants. We observed that changes in all *γ_i _*parameters reduce the final parasite load (see Figure 6). The rate constant that yields the minimum *for final, maximal and mean parasite load *is *γ_2 _*(after slightly increasing its value), which is the rate constant for parasite degradation (see Figure 1). Figure 6A shows final parasite load for different values of *γ_2_*. Figure 6B shows the effect on the parasite load for two increased values of *γ_2_*: 0.43 and 4.3.

### Combined two-parameter searches for identification of optimum parameter values

We carried out a systematic scanning of all the combinations of two parameters that yielded the minimum final parasite load. The rationale is that a combination of drugs makes it possible to reduce the parasite load in greater quantity, more quickly, and with lighter dosage than using only one drug. The search was limited to smaller parameter changes in the range of 60% - 180% of a parameter's original estimated value.

**Figure 7:**
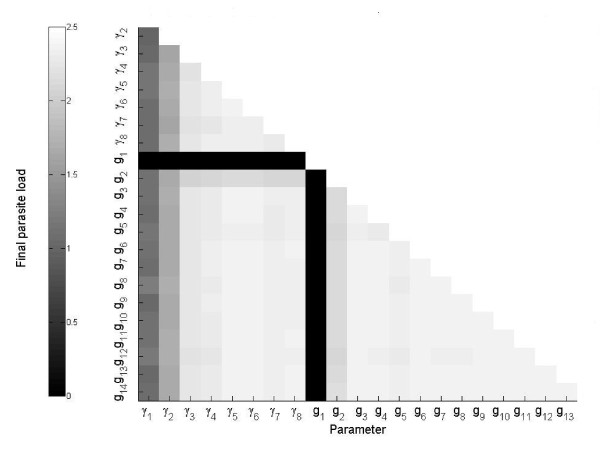
**Optimized final parasite load obtained for each possible combination of two parameters of the system**. The parasite load of the fitted system was 5.5.

We found that a total reduction of the observed final (as well as the maximal and mean) parasite load can be attained by simultaneously increasing g_1 _from its original value by a factor of 1.6 (approximately), and by changing any other of the remaining 21 parameters by different factors (Table 1). The other three variables (IgG1, IgG2a, lymphocytes) remain almost unchanged (results not shown). Figure 7 shows all possible combinations of two parameters and the optimum predicted final parasite load. Aside from the best combinations (see Figure 7, black column and file) g_1 _and g_2 _are also good choices for the minimization of parasite load. The remaining combinations also produce a reduction of parasite load, but to a lesser degree, and are considered to be of minor interest.

**Table 1:** Parameter change factors for the two parameter combinations involving g_1._

Parameter combination	FC(g_1_)/FCp_k_	Parameter combination	FC(g_1_)/FCp_k_
g_1_/γ_1_	1.73/0,67	g_1_/g_5_	1.77/0,81

g_1_/γ_2_	1.78/1,60	g_1_/g_6_	1.78/1,74

g_1_/γ_3_	1.79/1,20	g_1_/g_7_	1.78/1,49

g_1_/γ_4_	1.79/0,79	g_1_/g_8_	1.76/1,20

g_1_/γ_5_	1.78/1,26	g_1_/g_9_	1.77/1,18

g_1_/γ_6_	1.79/0,94	g_1_/g_10_	1.77/1,76

g_1_/γ_7_	1.79/1,20	g_1_/g_11_	1.79/1,58

g_1_/γ_8_	1.79/1,53	g_1_/g_12_	1.77/1,65

g_1_/g_2_	1.77/0,83	g_1_/g_13_	1.79/1,54

g_1_/g_3_	1.79/0,95	g_1_/g_14_	1.74/0,92

g_1_/g_4_	1.79/1,21		

The Parameter combination columns stand for the combination of parameter while the FC(g_1_)/FCp_k _columns indicate the ratio between the factor change (FC) of the original value or g1 over the FC of the corresponding parameter (p_k_)

By way of illustration, Table 1 show the parameter change factor for the two-parameter combinations involving g_1 _(black column and file in Figure 7). Interestingly, the solutions that lead to the lowest parasite loads have values for most parameters that are approximately 80% higher than their basal values. Exceptions include γ_1_, γ_4_, γ_6_, g_2_, g_3_, g_5_, and g_14_. Their values are reduced by factors ranging from 5 to 85% (see Table 1).

## Discussion

The standard leishmaniasis treatments are chemotherapy based, though some new treatments are based on the use of immunotherapy. In our model, the chemotherapeutical agents are those that target parasite destruction (g_2_) or inhibit proliferation (g_1_), whereas immunotherapeutic treatment implies changing parameters g_3_, ... g_8 _and g_3_, g_4_, g_6_, g_8_, g_9_, ...g_12_. In most cases the exact interaction mechanism of the drug is not yet known, though it is possible to associate them to the corresponding parameters that are being influenced. It is important to mention that if a given therapeutic agent has an influence that is not represented by any of our model's parameters but corresponds with the in- or outflux of a model variable, the effect of this agent can be translated in our model by a change in the respective rate constant γ_i_.

Regarding drug therapy, we have found three parameters which cause parasite load reduction: g_1_, which describes the influence of parasites on their own proliferation; g_6_, which represents the influence of lymphocytes on their own proliferation; and γ_2_, the rate constant for parasite degradation.

Examination of the standard drugs used for leishmaniasis treatment shows that most are aimed at parasite destruction. In our model that translates a an increase in γ_2_, the rate of parasite destruction [34], an observation that is coherent with our model's predictions. This is the case for several standard treatments such as amphotericin B (partially inhibits the completion of the parasite's membrane), antimonials (decrease biosynthesis of energy in the amastigote), and itraconazole and pyrazolopyrimidines (inhibit the parasite growth). Other substances currently under evaluation, such as betle leaves extract (reduces viability of promastigotes), interferon (actives macrophages that reduce parasite load), and IL-12 (stimulates Th1) also increase γ_2_. These observations constitute a pragmatic, *a posteriori *verification of our model's predictions.

Most of the therapeutic drugs used also seem to inhibit, albeit through different mechanisms, parasite proliferation: aminoglycosides alter parasite messenger RNA, pentamidine inhibits polyamine and DNA synthesis in the parasite, imidazole and itraconazole inhibit demethylation of membrane, and pyrazolopyrimidines block protein synthesis and destroy parasite RNA. All these effects can be interpreted, in terms of our model, as a decrease in g_1_. The discrepancy in our model's predictions can be explained by several facts. First, in all cases where a decrease in g_1 _could be assumed, there is also the concomitant effect of increasing γ_2_, as noted above. Thus, a trade-off of these two actions should be previously evaluated in order to have an accurate account of the whole drug effect. Second, it should be taken into account that the effect of a g_1 _modulation could be different depending on the stage of the disease. It has also been shown that if parasites replicate quickly, the immune system is able to recognize them more easily [13]. Parasites use mechanisms like inhibition of antigen presentation to escape immune response, however, a high growth rate induces massive macrophage recruitment [13]. At this point it should be stressed that our model considers the infection from the very initial stage. A third explanation could be that the parasites produce a certain molecule that stimulates an immune response of the body. (While investigating a model of tuberculosis infection [35], it was also found that the partial rank correlation between growth rate and extracellular bacteria is negative in a certain time interval.)

The factor that increases the influence of parasites on their own proliferation (g1) is crucial according to our model's results; and currently, no pharmaceuticals that increase g1 have been tested against Leishmania. Insuline-like growth factor 1, interferon, and possibly TNF-α cytikine could be considered as potential targets for stimulating parasite replication inside macrophages, and it would be of great interest to test their anti-leishmanial effectiveness. Insuline-like growth factor also increases the number of parasites (γ_1_) and reduces parasite-toxic production of nitric oxide (γ_2_).

Furthermore, no existing drug is known to have an effect on g_6_, which, in our analysis, is also seen as a possible effective pharmaceutical target. This clearly points to the new, potential application of existing and current therapeutic strategies.

The approach used for detecting key processes that must be regulated in order to reduce parasite load also allowed us to identify combinations of two drugs that would eventually be more effective than a single drug treatment. As is showed in Figure 7, combinations of drugs able to increase g_1 _or g_2 _and simultaneously change any other parameter, or, alternatively, combinations of drugs that decrease g_1 _together with the change in another parameter, would cause significant reduction in the final parasite load. These findings greatly amplify the number of therapeutic options available, although they still remain to be tested. By way of illustration, we could suggest the combination of any of the available drugs that increase g_2 _(amphotericin B, aminoglycosides, antimonials, pentamidine imidazole, itraconazole, and pyrazolopyrimidines) together with any of the following: interleukin-5,6,13 and MHC class II molecules (both increasing g_3_), rLmSTI1 (increase in g_4_), and chemokines (that increase g_3 _and g_2 _simultaneously). In the same mouse model we will test the effects on the variables of different drug combinations to verify the model's predictions and to eventually refine and extend the model by including new variables and mechanisms.

A limitation of the present approach is that our model is a simplification and does not include a detailed description of all the factors involved in the interaction mechanism of the drug in the body. However, given that these mechanisms are often not known, the modeling approach constitutes an approximation to the understanding of a complex dynamic system based on available information and informed hypothesis.

## Conclusions

In the present work we have illustrated a novel approach for the design of effective therapeutic strategies for leishmaniasis treatment. The approach is based on the integration of experimentally available information on infection development in an animal model using a mathematical model that describes the system dynamics observed. Many of the predictions concur with the standard practice, while others remain to be explored. We are confident that this rational, model-based approach is of great interest given that it overcomes the limitations of a trial and error strategy, and provides an extra layer of rationality in the search for new therapeutic formulas. This approach is also readily applicable to other parasitic-related illnesses.

## Methods

### Mice

BALB/c mice, 6-8 weeks old, were obtained from the animal breeding facilities of the Universidad de La Laguna. The experimental protocols used were approved by the Animal Care and Use Committee of the University of La Laguna (Approval ID number 132).

### Parasites and experimental infection of mice

Amastigotes of MPRO/BR/77/LTB0016 strain of *L. amazonensis *were prepared from infected BALB/c mice, retaining full virulence. Promastigotes derived from tissue amastigotes were then grown in Schneider's insect medium, pH 7.2, supplemented with 10% heat-inactivated fetal bovine serum, 100 U/ml penicillin and 100 μg/ml streptomycin at 26°C.

In order to follow up the evolution of the infection, 10^3 ^(10^6 ^in case of the verification experiments) stationary phase *L. amazonensis *promastigotes contained in 30 μl of PBS, were injected subcutaneously into the tarsi of right hind leg of 20 BALB/c. In 8 of the 20 mice the response of IgG1 and IgG2a antibodies was evaluated every 2 weeks during the 28-week period of study. The cellular response and parasite load were also measured, for which we had to sacrifice four mice at 8, 16 and 24 weeks of infection.

### Parasite quantitation

Estimates of the parasite number present in infected organs were done as described in Buffet et al. [36] which allowed for quantifying the parasite load from tissue homogenates.

Serial threefold dilutions ranging from 1 to 1/3 × 10^-6 ^were prepared twice for each homogenate in wells of 96-well plates, containing 200 μl of Schneider's insect medium, pH 7.2, supplemented with 10% heat-inactivated fetal bovine serum, 200 U/ml penicillin and 200 μg/ml streptomycin. After 7 and 10 days at 26°C, each well was examined and defined as positive or negative based on the presence or absence of viable promastigotes. A limiting dilution analysis was applied to the data to estimate the number of viable *Leishmania *expressed as Limiting Dilution Assay Unit. The total number of parasites per gram (parasite load) was calculated by equation 4, where *D+ *is the last dilution positive and *Po *is the weight of the piece of tissue.

(3) $$Parasite\;load=\frac{1∕D+}{Po}$$

### Antigen

The soluble antigen of the parasite (LaAgS) used for enzyme-linked immunosorbent assay (ELISA) determination and splenocytes proliferation assay was obtained from stationary phase cultures of MHOM/BR/77/LTB0016 strain promastigotes of *L. amazonensis *and according to Larreta et al. [37].

### Antibodies

The specific antibody response levels of IgG1 and IgG2a against LaAgS were determined by indirect ELISA in serum of BALB/c mice. ELISA assays were carried out using standard conditions. Microtiter plates were coated with 0.8 μg per well of the antigen. The sera from the mice were assayed at 1:80 dilutions. As secondary antibody the HRPO-conjugated goat anti-mouse IgG1 and IgG2a were used to 1:8000 and 1:1000, respectively. We used two groups of 8 mice, one experimental and one control.

### Lymphoproliferation

*In vitro *lymphoproliferation assays were carried out to measure the capacity of the LaAgS to stimulate the lymphocyte multiplication or proliferation, as indicative of the capacity of the parasite to produce a specific immune response. The lymphocytes were aseptically removed from spleens of experimental and control BALB/c mice and disrupted in PBS with 1% FCS. The cells were centrifuged, the erythrocytes were lysed in a lysis solution (150 mM NH_4_Cl, 10 mM KHCO_3_, 1 mM EDTA, pH 7.4) and remaining cells were finally resuspended to a density of 2.5 × 10^6 ^cells/ml in DMEM containing 10% FCS, 2 mM L-glutamine, 0.05 mM 2-mercaptoethanol, 12 mM HEPES, pH 7.1, 100 IU/ml penicillin and 100 μg/ml streptomycin. Lymphocytes were divided into 100 μl aliquots (2.5 × 10^5 ^cells) in 96-well plates and they were allowed to proliferate for 3 days at 37°C in an atmosphere containing 5% CO_2 _and 95% humidity in the presence or absence of LaAgS (final concentration 40 μg/ml).

Proliferation was measured by SRB assay, following the protocol by Monks et al. [27]. Absorbance due to the incorporation of the SRB coloring to anionic proteins of viable lymphocytes, as an index of proliferation stimulation, was measured using a micro plate reader at 570 nm (Model 680; BIO-RAD). Stimulation index of the lymphocyte proliferation (SI) of experimental mice was calculated according the following equation:

(4) $$SI=\frac{\bar{X}AbsTw-\bar{X}AbsCw}{Cutoff}$$

where $\bar{X}$*Abs_Tw _*and $\bar{X}$*Abs_Cw _*stand for the average absorbance value for the antigen treated wells and the average of the average absorbance value for the control wells (only DMEM), respectively. The *Cutoff *point was calculated as the average difference between the treated wells absorbance minus the control wells absorbance plus 3 SD (standard deviation) of the absorbance of lymphocytes of control mice. The *Cutoff *was established for each assay. We used two groups of 12 mice, one experimental and one control. All assays were performed in triplicate with four mice representing each group.

### Parameter estimation

In the power-law model used, the parameters of the model, kinetic orders (g_i_) and rate constants (γ_i_) were estimated from experimental data using a genetic algorithm adapted for power-law models [22,38]. The initial population of solutions is generated through a random exploration of the search space, which is defined using feasible intervals of values for the parameters. The best individuals of the population are selected in the considered iteration based on the value of the following objective function:

(5) $$F_{Obj}=\frac{\text{1}}{n_{\text{var}}}\overset{n_{\text{var}}}{\underset{j=\text{1}}{\sum }}\overset{n_{tp}}{\underset{i=\text{1}}{\sum }}\frac{{\left(X_{j}\left(t_{i}\right)-X_{j}^{\text{exp}}\left(t_{i}\right)\right)}^{\text{2}}}{n_{tp}}$$

where *n_var _*is the number of variables monitored and *n_tp _*the number of time points when each variable was measured. In turn, X_j_(t_i_) is the predicted value for the *j^th ^*variable at the *i^th ^*time point obtained after numerical integration of the solution, while X_j_^exp^(t_i_) is the value of the *j^th ^*observable variable at the *i^th ^*time point measured in the experiment. This objective function has been tested in other case studies [22,38] showing that it is independent of time and therefore will not be affected by the measurements schedule. We consider it to be well suited for this type of fitting problem. The objective function takes into account the 24-week period for which there is available experimental data of all variables; in the fitting process all variables are weighted equally. The fitting process stops if either the objective function converges below a threshold value or the maximum number of iterations is exceeded. The first criterion is fulfilled when it reaches objective function values smaller than a previously defined one. The second occurs when it reaches a certain number of generations. However, it is possible to set up a combination of both so that parameter estimation stops when a solution with an objective function value below a predetermined parameter is reached, or a certain number of generations have been explored. Every few generations a controllable number of individuals of the total population are selected, and the objective function-defined surface is submitted to local scrutiny taking as starting points selected individuals. For each starting point, parameters are changed in a small and random amount and the new objective function is evaluated. If the new value is lower than its initial value, the new set of parameters is adopted, otherwise the original parameters are kept. In our present case we used a combination stopping criterion, namely 1000 generations, as the maximal number of iterations, and an objective function value smaller than 0.2.

Parameter estimation was executed from the previously obtained time series of experimental data after normalization [39]. All γ_i _were permitted to vary within the range [0,10] and the g_i _within the range [0,3], except the parameters g_2_, g_8_, g_11 _and g_14 _which were set to 1 since they relate variables with their own outflow (V_2_, V_4_, V_6 _and V_8_, respectively). This model hypothesis is a biologically plausible one that permits reduction in terms of the number of parameters. These ranges come from physiological as well as kinetic considerations. Processes in which kinetic orders are greater than 3 are rarely observed since this corresponds to processes or reactions with extreme sensitivity to changes in one of the reactants. A similar reasoning applies to higher γ_i _values, although in this case the admissible range is wider. However, in the optimization search we used greater ranges. The rationale is that in the optimization search we can assume possible wider ranges as physiologically acceptable because the optimized system would correspond with altered kinetic behavior with the use of drugs or other therapeutic agents.

Parameter estimation using 1000 iterations which required a computation time of 96 hours. The objective function value finally reached was 0.0517, with a maximal absolute error between interpolated data and model of 0.5828. In accordance with the objective function definition, this measures the average distance between the experimental data and the model. Since the standardized experimental variation values range between 0 and 2.5, an objective function value of 0.0517 means an average relative error lower than 5%, which is less than the experimental error and therefore enough to ensure that the model represents the experimental data.

All variables in the model are normalized. Parasite load is normalized with respect to its proper mean and the remaining variables are normalized with respect to the control group of mice. This standardization reduces the range of variation of the parameters and computation time and also exploits various properties of the GMA-PL formalism concerning the behavior of variables and parameters. Initial values of parasite load between 10^-10 ^and 0.01 were implemented, comparing them in terms of the objective function. The smaller the minimum of the objective function, the better the model (with the respective initial value) fits the experimental data. Of the initial values for which simulations meet the criterion f_obj _< 0.2, we found that the best fit and lowest objective function was attained with 10^-6 ^as the initial value for parasite load in the group infected with 10^3 ^parasites. Therefore 10^-6 ^was used as the initial value in that group.

### Model selection strategies

The topology of the model is assumed to be as it appears in Figure 1. There are different models according to the number of parameters used; the most general model has 22 parameters. In other simplified models the value of certain parameters were assumed to be zero or one [21]. Among the different strategies of model selection for finding the one that yielded the "best" fit, we finally chose to specify a fixed value of the objective function and, accordingly, selected models that fell below this value. Out of these, we chose the simplest model, that is, the model with the least number of parameters.

### Dynamic Sensitivities

Sensitivity analysis is a tool useful for model robustness evaluation and system dynamics characterization. Since our model studies the system dynamics, this tool enables us to identify the parameters with major influence on the transient dynamics. We have used the System Parameter Dynamic Sensitivity, S*_pk_*^Xi^, defined in equation 6:

(6) $$S_{pk}^{Xi}\left(X_{i},p_{k}\right)=\text{sgn}\;\left(\frac{A_{k,2}-A_{k}}{p_{k,2}-p_{k}}\right)\frac{D_{k}}{\frac{\left|p_{k,2}-p_{k}\right|}{p_{k}}}$$

In the above expression, *Xi *is the considered variable, while *pk *is the parameter under scrutiny. A*k,2 *is the area below the solution curve after a change of *pk *to *pk,2*. A*k *is the area under the solution curve using the original value *pk*. D*k *is the area between the two solution curves, using *pk *and *pk,2*, respectively. In our analysis we have considered changes in the two kinds of parameters, kinetic orders and rate constants. The S*_pk_*^Xi ^value corresponds to the variation of the area under the variable time course after perturbation in parameter space. For each model variable the absolute values of the area *A_k _*between the original curve and the abscissa, the area *A_k2 _*between the new curve and the abscissa and the area *D_k _*between the original and the new curve are calculated using the trapezoidal method. Positive sensitivity means that the area under the "new" curve is greater than the area under the original curve, i.e., that the parameter *pk *has a positive influence on variable Xi. Negative sensitivity means the opposite. Zero sensitivity means that small changes in the parameter have no influence on the variable. All sensitivities were computed for standardized variables.

## Authors' contributions

BV and NVT proposed the elaboration of a mathematical model based on the GMA power-law formalism of the progression of the disease and with the potential to be used for the design of effective therapies. They planned the work and coordinated the study. CP-B carried out parasite, antigen, antibodies and lymphoproliferation quantization. BML implemented the GMA-PL model.

BML, BW and CG-A performed the optimum parameter searches. NVT, BV, BML and CP-B and CG-A defined the model and obtained the numerical parameters used in the paper. All authors read and approved the final manuscript.
